# Factors Influencing eHealth Literacy Worldwide: Systematic Review and Meta-Analysis

**DOI:** 10.2196/50313

**Published:** 2025-03-10

**Authors:** Zhong Hua, Song Yuqing, Liu Qianwen, Chen Hong

**Affiliations:** 1 Department of Nursing West China School of Nursing, West China Hospital Sichuan University Chengdu China; 2 Department of Gynecological Nursing West China Second University Hospital Sichuan University Chengdu China; 3 Key Laboratory of Birth Defects and Related Diseases of Women and Children (Sichuan University) Ministry of Education Chengdu China

**Keywords:** meta-analysis, eHealth literacy, eHealth, Technology Acceptance Model, Literacy and Health Conceptual Framework, social determinants of health, digital health, consumer

## Abstract

**Background:**

eHealth literacy has increasingly emerged as a critical determinant of health, highlighting the importance of identifying its influencing factors; however, these factors remain unclear. Numerous studies have explored this concept across various populations, presenting an opportunity for a systematic review and synthesis of the existing evidence to better understand eHealth literacy and its key determinants.

**Objective:**

This study aimed to provide a systematic review of factors influencing eHealth literacy and to examine their impact across different populations.

**Methods:**

We conducted a comprehensive search of papers from PubMed, CNKI, Embase, Web of Science, Cochrane Library, CINAHL, and MEDLINE databases from inception to April 11, 2023. We included all those studies that reported the eHealth literacy status measured with the eHealth Literacy Scale (eHEALS). Methodological validity was assessed with the standardized Joanna Briggs Institute (JBI) critical appraisal tool prepared for cross-sectional studies. Meta-analytic techniques were used to calculate the pooled standardized β coefficient with 95% CIs, while heterogeneity was assessed using I2, the Q test, and τ2. Meta-regressions were used to explore the effect of potential moderators, including participants’ characteristics, internet use measured by time or frequency, and country development status. Predictors of eHealth literacy were integrated according to the Literacy and Health Conceptual Framework and the Technology Acceptance Model (TAM).

**Results:**

In total, 17 studies met the inclusion criteria for the meta-analysis. Key factors influencing higher eHealth literacy were identified and classified into 3 themes: (1) actions (internet usage: β=0.14, 95% CI 0.102-0.182, I2=80.4%), (2) determinants (age: β=–0.042, 95% CI –0.071 to –0.020, I2=80.3%; ethnicity: β=–2.613, 95% CI –4.114 to –1.112, I2=80.2%; income: β=0.206, 95% CI 0.059-0.354, I2=64.6%; employment status: β=–1.629, 95% CI –2.323 to –0.953, I2=99.7%; education: β=0.154, 95% CI 0.101-0.208, I2=58.2%; perceived usefulness: β=0.832, 95% CI 0.131-1.522, I2=68.3%; and self-efficacy: β=0.239, 95% CI 0.129-0.349, I2=0.0%), and (3) health status factor (disease: β=–0.177, 95% CI –0.298 to –0.055, I2=26.9%).

**Conclusions:**

This systematic review, guided by the Literacy and Health Conceptual Framework model, identified key factors influencing eHealth literacy across 3 dimensions: actions (internet usage), determinants (age, ethnicity, income, employment status, education, perceived usefulness, and self-efficacy), and health status (disease). These findings provide valuable guidance for designing interventions to enhance eHealth literacy.

**Trial Registration:**

PROSPERO CRD42022383384; https://www.crd.york.ac.uk/PROSPERO/view/CRD42022383384

## Introduction

The links between literacy and health have attracted increasing international attention over the past decade [1]. Numerous studies have demonstrated that health outcomes, such as health behaviors and disease risks, are influenced by various forms of literacy, including health literacy, eHealth literacy, and other related literacy [2]. Especially in an era characterized by advanced information technology, the importance of eHealth literacy cannot be more pronounced due to the widespread adoption of digital health applications. eHealth literacy is defined as the capacity to search for, access, and analyze health information from electronic resources to solve health problems [3]. According to the Lily model of eHealth literacy, the 6 core literacies of eHealth literacy encompass essential aspects of health literacy and digital information literacy, including traditional literacy, health literacy, information literacy, scientific literacy, media literacy, and computer literacy [3]. Therefore, eHealth literacy not only directly influences the adoption of eHealth by users [3] but also is founded as a new determinant of health [4].

Studies have also found that eHealth literacy is positively associated with different aspects of health promotion and care. It influences an individual’s acquisition, judgment, and application of health knowledge [5]. It also shapes their perception and attitude toward health and disease [6,7], especially in an era of advanced social media. eHealth literacy also plays a facilitating role in promoting the positive adoption of health-promoting behaviors [8-10]. Ultimately, this has a positive impact on health-related physical, behavioral, psychosocial, and cognitive outcomes [2,11,12]. Thus, understanding the determinants of eHealth literacy to identify intervention targets is of significant research value. Such efforts can enhance patients’ eHealth literacy levels, ultimately contributing to improved health outcomes.

It is crucial for health care professionals to assess an individual’s eHealth literacy before developing and conducting target interventions. Some tools have been developed to measure eHealth literacy. These include the eHealth Literacy Scale (eHEALS), the eHealth Literacy Questionnaire (eHLQ) [13], and the Digital Health Literacy Instrument (DHLI) [14]. eHEALS, developed by Norman and Skinner [15], is the most widely used scale to assess eHealth literacy. It comprises 8 items and encompasses 3 dimensions (application, evaluation, and decision-making capabilities related to online health information and services). eHEALS has been translated into different languages and validated as exhibiting robust reliability and validity. Additionally, numerous studies have evaluated individuals’ eHealth literacy. However, the findings have not been systematically summarized. Therefore, this review aimed to synthesize the findings of studies that have used eHEALS to assess eHealth literacy. Although many studies have explored eHealth literacy among various populations, such as students, older adults, and the general population, the results have been inconsistent. Additionally, the influencing factors of eHealth literacy are diverse, including sociodemographic factors (ie, age, gender, and educational level), health-related behaviors, and internet usage. Despite the large number of cross-sectional surveys conducted to explore these factors, complex influences from both micro and macro levels have been identified, with results varying significantly [16]. The Literacy and Health Conceptual Framework, developed by the Literacy and Health Research Program in Canada, outlines 4 key themes: actions, determinants, literacy, and effects of literacy. Actions (eg, policy, training) and determinants (eg, sociodemographic status) can influence literacy (eg, general, health, and eHealth literacy). The Technology Acceptance Model (TAM) is a widely used theoretical framework. TAM posits that perceived usefulness and perceived ease of use directly influence an individual’s attitude toward using a technology, in turn, affecting their intention to use the technology. TAM has been widely used to explore the user acceptance of various information systems and technologies. Thus, the Literacy and Health Conceptual Framework [1] and TAM can serve as theoretical frameworks to summarize and integrate the influencing factors of eHealth literacy [17,18].

Consequently, we systematically reviewed studies on eHealth literacy among different populations and synthesized their findings through a meta-analysis to understand the eHealth literacy status and examine its influencing factors.

## Methods

### Overview

This systematic literature review was conducted in accordance with the Cochrane Collaboration [19] and PRISMA (Preferred Reporting Items for Systematic Reviews and Meta-Analyses) guidelines [20] (Multimedia Appendix 1). The study was registered in PROSPERO (International Prospective Register of Systematic Reviews; ID CRD42022383384).

### Search Strategy

We searched PubMed, CNKI, Embase, Web of Science, Cochrane Library, CINAHL, and MEDLINE databases from inception to April 11, 2023, to obtain a preliminary list of relevant studies. To develop search strategies, we checked 2 main sets of keywords: (1) eHealth literacy and corresponding synonyms (digital health literacy, electronic health literacy, e-health literacy, online health literacy, etc) and (2) influencing factors and corresponding synonyms. For both sets of keywords, we also reviewed the thesaurus and Medical Subject Headings (MeSH) terms. Next, we developed a search strategy for each database. An expert in information retrieval was consulted during this process. After formulating the initial search strategy, we conducted a preliminary search for each database. The search terms and results for each database were then sent to the expert for confirmation and refinement, ensuring the accuracy and comprehensiveness of the search strategy across all databases. The detailed strategies for the different databases are presented in Multimedia Appendix 2. These search terms were used to search titles and abstracts in all the selected databases without applying any filters or limits.

### Eligibility Criteria and Study Selection

We included all studies that were written in English or Chinese and reported the eHealth literacy status measured with eHEALS. For influencing factor analysis, we excluded (1) studies that did not examine the relationship between influencing factors and eHealth literacy or included only univariate analysis; (2) studies that did not provide effect size indicator values in the results or values that could not be converted to β, SE, and 95% CI values; and (3) reviews, case studies, poster presentations, and conference presentations. However, we examined their references to identify additional relevant papers for inclusion. We also manually searched the reference lists of studies in the final sample for additional relevant papers.

### Data Extraction and Management

Two reviewers (authors HZ and QL) independently screened each identified study in duplicate for inclusion, initially reviewing the title and abstract, followed by a full-text review, as necessary. All discrepancies were resolved through consensus. We extracted the following data from each study: country, year conducted, sample size, questionnaire used to measure eHealth literacy, status of eHealth literacy, influencing factors with details, and effect size values. Where relevant data were missing or incomplete, we contacted the authors for clarification and verification.

### Critical Appraisal of Methodological Quality

The included studies were critically appraised for methodological quality using the standardized Joanna Briggs Institute (JBI) critical appraisal tool prepared for cross-sectional studies and cohort studies [21] (Multimedia Appendix 3). Each study was evaluated by 2 independent assessors.

### Data Analysis

#### Descriptive Statistics and Narrative Synthesis of the Studies in the Final Sample

Descriptive statistics were used to summarize the characteristics of the included studies. Narrative synthesis was used to synthesize the findings related to eHealth literacy in the studies, for which means (SDs) were reported. The year, country, sample size, study population, mean age (SD) of the sample, funding, mean score (SD) of eHEALS, and influencing factors were examined.

#### Random-Effects Meta-Analyses and Fixed-Effects Meta-Analyses to Examine Factors Affecting eHealth Literacy

Meta-analyses were conducted separately for eHealth literacy and each influencing factor using StataMP 17 software. Pooling was considered when at least 2 studies assessed eHealth literacy and its influencing factors. Standardized β coefficient estimates and their 95% CIs were calculated using random- or fixed-effects meta-analysis based on I^2^ and the number of studies [22,23]. If *I*^2^≤50%, the study was considered homogeneous and a fixed-effects model was used for meta-analysis. If *I*^2^>50%, the random-effects model was selected. If *I*^2^>85% and the source of heterogeneity could not be determined, meta-analysis was not performed and descriptive analysis was used. In addition, the fixed effects model was considered if the number of studies that reported eHealth literacy’s influencing factors was small (<5) [23,24]. Forest plots were created to show the results, and all comparisons were 2-tailed using a threshold of *P*≤.05.

The heterogeneity of effect sizes was determined using the following statistics: (1) *Q* test of heterogeneity, (2) *τ*^2^ estimate of true between-study variance, and (3) *I*^2^ statistic of the proportion of true variation in observed effects. The Egger test was used to assess the possibility of publication bias [25].

#### Meta-Regression Analysis to Examine the Cofounders Affecting eHealth Literacy

The effect of moderators (participants’ characteristics, internet use measured by time or frequency, country development status) was measured via random-effects univariate meta-regressions using restricted maximum-likelihood estimation. For the analysis, participants were categorized into 3 groups: students (coded as 1), the general population (coded as 2), and older adults (coded as 3). Regarding the country variable, studies conducted in low- to middle-income countries were coded as 1, while those conducted in high-income countries were coded as 2. The development status of a country was queried on the United Nations Big Data Global Platform [26]. To conduct cofounder analyses and obtain robust coefficient estimates, we followed the recommendations of Fu et al [27] and conducted moderator analysis only when at least 4 studies per group were available.

#### Assessment of the Quality of Evidence

The quality of evidence for the results of the meta-analyses was evaluated by authors QL and HZ using the GRADE (Grading of Recommendations, Assessment, Development, and Evaluation) framework [28]. For each factor influencing eHealth literacy, the initial quality of evidence was considered high and was downgraded by 1 level for every significant concern identified within the domains of risk of bias, inconsistency, indirectness, imprecision, and publication bias. The risk of bias was evaluated by examining potential biases in participant selection, measurement instruments, data collection, and analysis. The inconsistency domain was assessed using *I*^2^ values, and the GRADE quality was downgraded when *I*^2^ was 50% or higher. Indirectness was evaluated based on whether the studies directly assessed the influence of various factors on eHealth literacy. Imprecision was determined by the width of the CIs of the estimates and the sample size. Publication bias was assessed using the Egger test, with GRADE quality being downgraded for statistically significant results (*P*<.05) in this test.

### Theoretical Support for the Integration Framework of Influencing Factors

**Figure 1:**
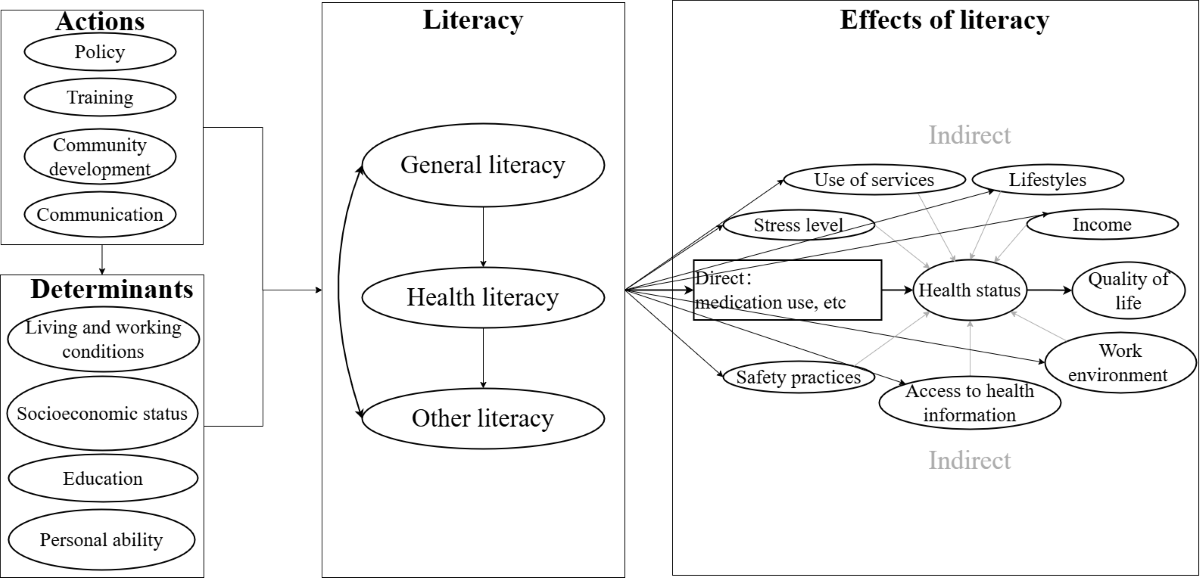
The Literacy and Health Conceptual Framework.

**Figure 2:**
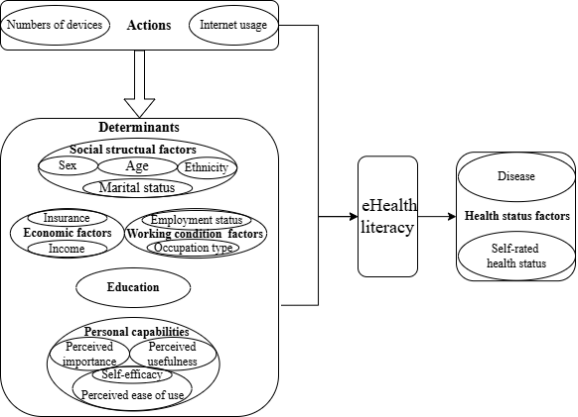
Factors influencing eHealth literacy in our analysis.

This study combined the Literacy and Health Conceptual Framework [1] and TAM [17,18] to synthesize and present factors that influence eHealth literacy, as identified in the literature review. The Literacy and Health Conceptual Framework (Figure 1), developed through the Literacy and Health Research Program sponsored by the Social Sciences and Humanities Research Council of Canada, comprises 4 key themes: actions, determinants, literacy, and effects of literacy. Actions and determinants can impact literacy and the effects of literacy. Each theme is further subdivided into various conceptual elements that have been delineated in greater detail. Arrows and line segments within the framework illustrate the interconnections and relationships among these elements. Actions include policy, training, community development, and communication. Determinants comprise sociodemographic status, living and working status, education, and personal ability. Literacy comprises health literacy, general literacy, and other literacy. In our study, we used the Literacy and Health Conceptual Framework as the primary structure to organize all influencing factors (Figure 2). We identified literacy as eHealth literacy and summarized its influencing factors into actions and determinants.

TAM, a theoretical framework developed by Venkatesh and coworkers [17,18], predicts individuals’ adoption of new information technologies and has been extensively applied in eHealth domains. TAM posits that perceived usefulness and perceived ease of use mediate individuals’ intention to use technology by shaping their attitudes toward it. The third iteration of this model, TAM3, expands on the original by integrating additional components, such as computer self-efficacy, which reflects an individual’s confidence in their ability to perform tasks using a computer. Studies have found that perceived importance is strongly associated with both perceived usefulness and eHealth literacy [29-31]. In our study, we categorized key aspects of TAM3, including perceived usefulness, perceived importance, and self-efficacy, under personal capabilities. This emphasized that technology acceptance abilities are part of an individual’s overall capabilities (Figure 1).

## Results

### Literature Selection Process and Characteristics of the Final Studies

**Figure 3:**
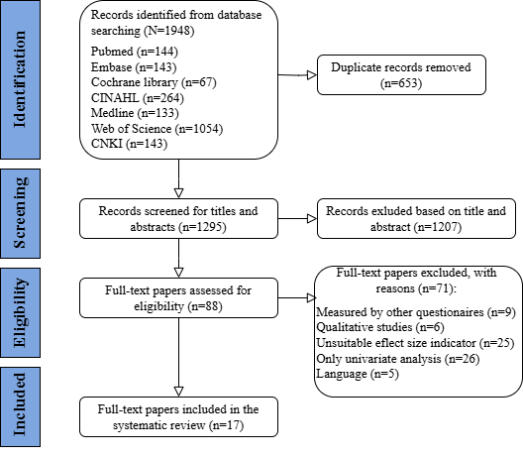
PRISMA flow diagram of the study selection process. eHEALS: eHealth Literacy Scale; PRISMA: Preferred Reporting Items for Systematic Reviews and Meta-Analyses.

The process of literature search and selection is shown in Figure 3. In total, 1295 potential papers were identified, but only 17 (1.3%) were identified as eligible and included in the final review to analyze the relationship between eHealth literacy and influencing factors (Figure 2). All the studies were cross-sectional in design (Table 1 and Table S1 in Multimedia Appendix 4). They were conducted in Turkey (n=1, 6%), China (n=1, 6%), Italy (n=1, 6%), South Korea (n=2, 12%), Serbia (n=1, 6%), Sweden (n=1, 6%), Australia (n=1, 6%), Vietnam (n=1, 6%), Kuwait (n=1, 6%), Saudi Arabia (n=1, 6%), Thailand (n=2, 12%), the United States (n=1, 6%), Malaysia (n=1, 6%), the United Kingdom (n=1, 6%), and Portugal (n=1, 6%). The study samples were the general population (n=3, 17.6%), older adults (n=3, 17.6%), nursing students (n=2, 12%), adult residents of urban and rural communities (n=1, 6%), undergraduate and postgraduate students (n=1, 6%), only undergraduate students (n=1, 6%), middle school students (n=1, 6%), adolescents (n=1, 6%), adults (n=1, 6%), medical students (n=2, 12%), and army personnel (n=1, 6%). Only 10 (59%) studies reported the age of participants. The mean age of students ranged from 20.13 (SD 2.16) to 24 (SD 1.48) years, and the mean age of adults ranged from 47 (SD not reported) to 78 (SD 2.4) years. All the studies were published in 2015 and later, and the majority (n=14, 82%) were published between 2018 and 2023. In addition, 15 (88%) studies had sample sizes greater than 100.

**Table 1:** Details of the 17 studies included in this meta-analysis.

Study	Country	Study population	Age (years), mean (SD)	eHealth literacy status^a^, mean (SD)
Tennant et al [9]	United States	Older adults	67.46 (9.98)	29.05 (5.75)
Alhuwail and Abdulsalam [29]	Kuwait	General population	NR^b^	28.63 (5.6)
Sinan et al [31]	Turkey	Nursing students at nursing departments of 2 state universities in Ankara, Turkey	21.14 (1.62)	29.28 (4.73)
Zuo et al [32]	China	Adult residents of urban and rural communities	NR	32 (24.40)^c^, 28 (21.34)^d^
Del Giudice et al [33]	Italy	Undergraduate and postgraduate students	NR	28.2 (6.2)
Park [34]	South Korea	Middle school students	NR	28.72 (5.68)
Gazibara et al [35]	Serbia	Adolescents (14-19 years)	NR	26.0 (10.0)
Ghazi et al [36]	Sweden	Older adult participants of the Swedish National Study on Aging and Care, Blekinge (SNAC-B)	78 (2.4)	27.5 (10.8)
Richtering et al [37]	Australia	Adults with moderate-to-high risk for a cardiovascular disease event	67 (8.0)	27.2 (6.59)
Hoang Nguyen and Bich Thi Le [38]	Vietnam	Medical students	NR	27.03 (3.54)
Almoajel et al [39]	Saudi Arabia	General population	24 (1.48)	28.79 (6.75)
Wongjinda and Taneepanichsakul [40]	Thailand	Royal Thai Army (RTA) personnel	NR	31.6 (NR)
Lee et al [41]	Malaysia	General population	47 (NR)	27.38 (6.59)
Kim and Jeon [42]	South Korea	Nursing students	21.69 (2.77)	29.68 (NR)
Holch and Marwood [43]	United Kingdom	Undergraduate students	20.13 (2.16)	29.46 (4.91)
Tanasombatkul et al [44]	Thailand	Medical students	22 (NR)	33.45 (3.28)
Martins et al [45]	Portugal	Older adults	67.36 (7.23)	NR

^a^Measured with the eHealth Literacy Scale (eHEALS).

^b^NR: not reported.

^c^Median (IQR) of eHEALS for rural community residents.

^d^Median (IQR) of eHEALS for urban community residents.

### Quality of Included Studies

Based on the JBI critical appraisal tool, the overall quality of the included studies was high. Of the 17 studies, 6 (35%) achieved a perfect score of 100%, 7 (41%) scored 87.5%, 3 (18%) scored 75%, and only 1 (5%) scored 62.5% (Multimedia Appendix 3). This result demonstrated that the majority of the included studies (n=13, 76%) were rated as high-quality studies, with scores of 75% or higher [46].

### Influencing Factors

According to the Literacy and Health Conceptual Framework [1], the factors influencing eHealth literacy were categorized into 3 themes: actions, determinants, and health status (Figure 2). Perceived importance, perceived usefulness, and self-efficacy, as defined by TAM3 [17], were integrated under the broader concept of personal capabilities. All the outcomes of the meta-analyses are presented in Table 2.

**Table 2:** Results of the analysis of influencing factors.

Theme and influencing factors			Studies (N=17), n (%); sample size, N		β (95% CI)		*I*^2^ (%)		Egger test *P* value		GRADE^a^ quality of evidence
**Actions**											
	Number of devices	3 (18); 781		0.172 (–0.460 to 0.805)		0		.35		Moderate	
	Internet usage	10 (59); 4243		0.141 (0.102 to 0.182)^b^		80.4		.56		Low	
**Determinants:** **social determinants of health**											
	Age	13 (76); 4330		–0.042 (–0.071 to –0.020)^b^		80.3		.18		Low	
	Ethnicity	2 (12); 559		–2.613 (–4.114 to –1.112)^b^		80.2		N/A^c^		Very low	
	Marital status	4 (24); 1535		–0.143 (–0.354 to 0.069)		27.4		.84		Moderate	
	Sex	8 (47); 2812		–0.134(–0.561 to 0.304)		64.6		.30		Moderate	
**Determinants:** **economic and working conditions**											
	Income	6 (35); 1997		0.206 (0.059 to 0.354)^b^		64.6		.30		Moderate	
	Insurance	2 (12); 789		–0.007 (–0.199 to 0.184)		0		N/A		Moderate	
	Employment status	2 (12); 612		–1.629 (–2.323 to –0.953)^b^		99.7		N/A		Very low	
	Occupation type	2 (12); 916		–0.242 (–0.644 to 0.160)		42.7		N/A		Moderate	
**Determinants:** **personal capabilities**											
	Perceived importance	3 (18); 1745		0.563 (–0.198 to 1.325)		58.1		.14		Moderate	
	Perceived usefulness	2 (12); 686		0.832 (0.131 to 1.522)^b^		68.3		N/A		Low	
	Self-efficacy	2 (12); 393		0.239 (0.129 to 0.349)^b^		0		N/A		Moderate	
Determinants: education			16 (94); 6331		0.154 (0.101 to 0.208)^b^		58.2		.86		Moderate
**Health status**											
	Self-rated health status	3 (18); 1451		0.564 (–0.198 to 1.323)		17.3		.47		Moderate	
	Disease	7 (41); 2817		–0.177 (–0.298 to –0.055)^b^		26.9		.29		High	

^a^GRADE: Grading of Recommendations, Assessment, Development, and Evaluation.

^b^The factor was significant in the meta-analysis.

^c^N/A: not applicable (as <3 experiments were analyzed).

#### Influencing Factors in the Actions Theme

**Figure 4:**
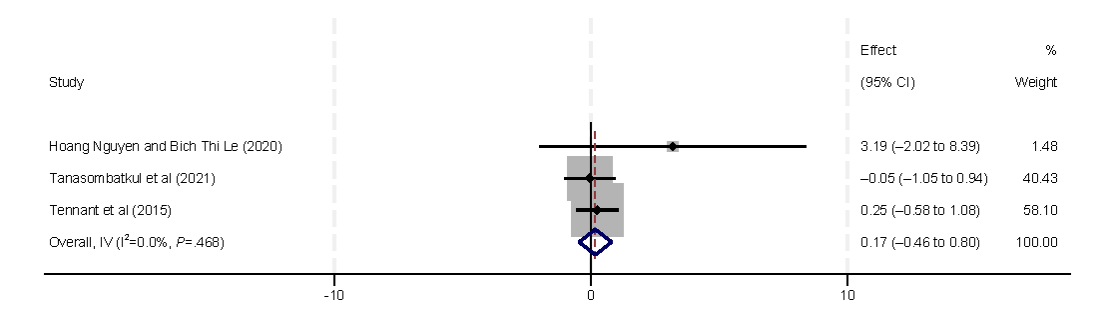
Forest plot of the meta-analytic association between the number of devices and eHealth literacy. IV: inverse variance weighted.

The influencing factors in actions included the number of devices and internet usage. Three studies [9,38,44] identified an association between eHealth literacy and the number of devices (Figure 4). These studies examined whether the number of electronic devices an individual owns is correlated with their eHealth literacy. However, the results of our analysis did not yield any statistically significant findings. Eight studies [29,31,33,37,40,42,44,45] demonstrated a positive correlation between eHealth literacy and internet usage (β=0.14, 95% CI 0.10-0.18).

**Figure 5:**
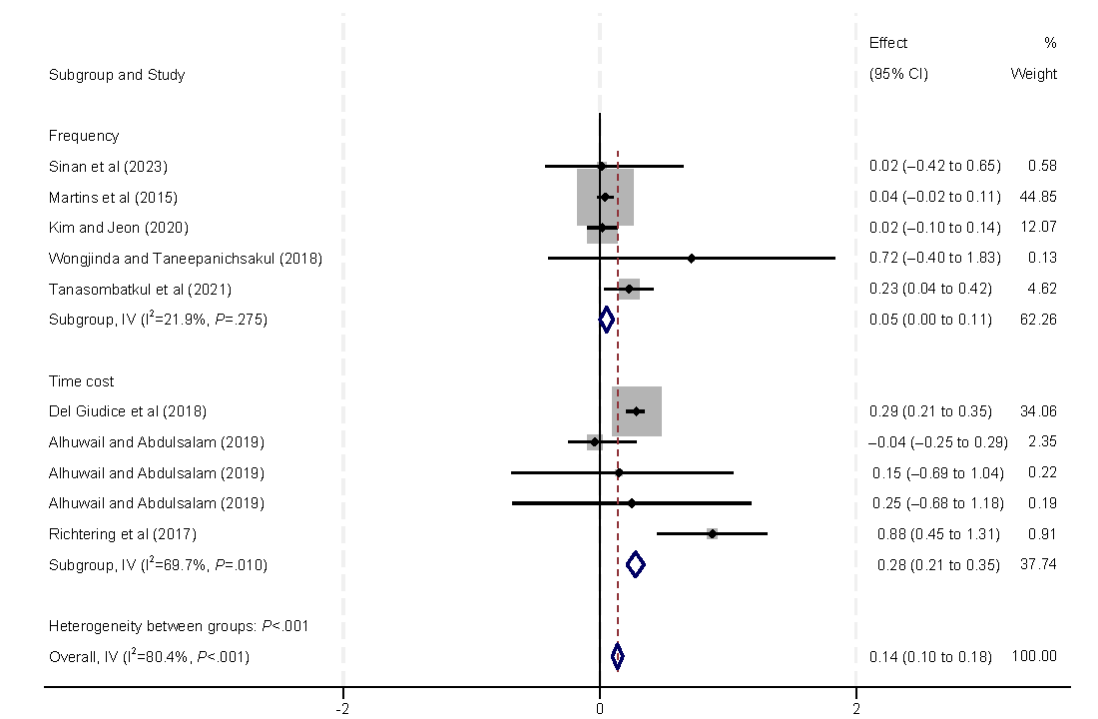
Forest plot of the meta-analytic association between internet usage and eHealth literacy. IV: inverse variance weighted.

Furthermore, we conducted subgroup analyses to examine internet use measured by duration or frequency (Figure 5). The results from both subgroups showed a positive correlation between internet usage and eHealth literacy, indicating that higher levels of internet use are associated with increased eHealth literacy.

#### Influencing Factors in the Determinants Theme

Social determinants of health, economic and working conditions, education, and personal capabilities were included under the determinants theme.

##### Social Determinants of Health

**Figure 6:**
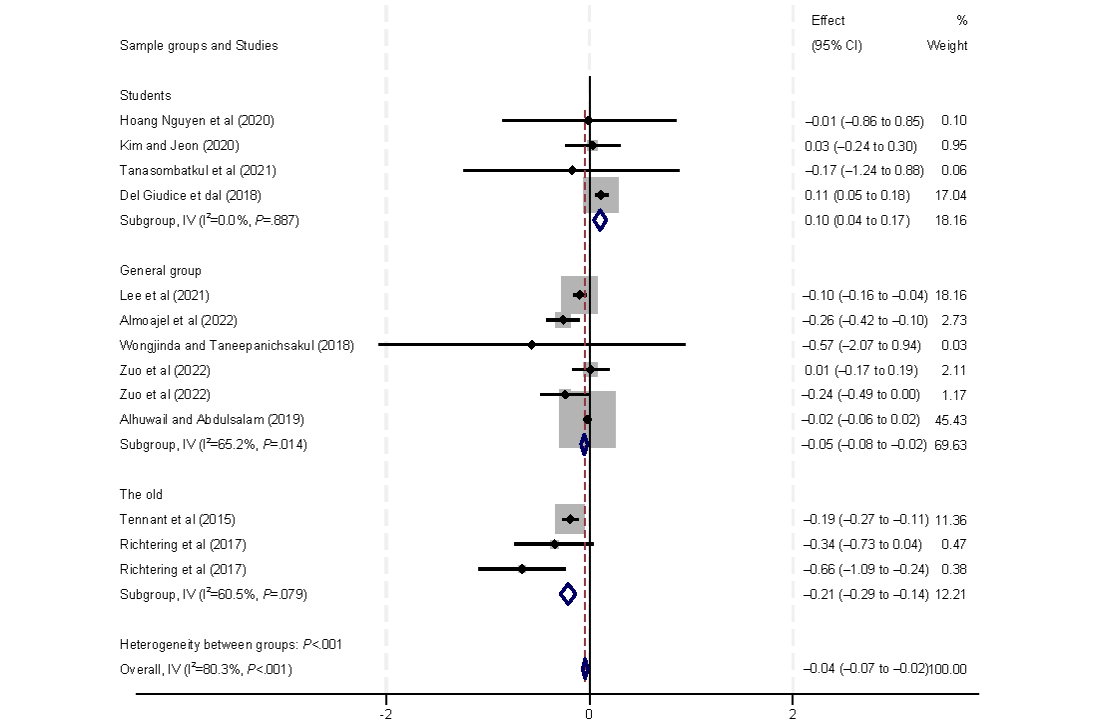
Forest plot of the meta-analytic association between age and eHealth literacy. IV: inverse variance weighted.

The following social determinants of health were analyzed to assess their relationship with eHealth literacy: age, ethnicity, marital status, and sex. The age of participants was significantly associated with eHealth literacy (Figure 6). Only 1 (13%) [33] of the 8 (47%) studies assessing eHealth literacy found a positive correlation with age, with the other 9 (53%) studies [9,29,37-42,44] reporting a negative correlation. Subgroup analyses based on age revealed that studies conducted among students showed a positive correlation between eHealth literacy and age (β=0.10, 95% CI 0.04 to –0.17). Conversely, in the general population (β=–0.05, 95% CI –0.08 to –0.02) and among older adults (β=–0.21, 95% CI –0.29 to –0.14), eHealth literacy was found to be negatively correlated with age.

**Figure 7:**
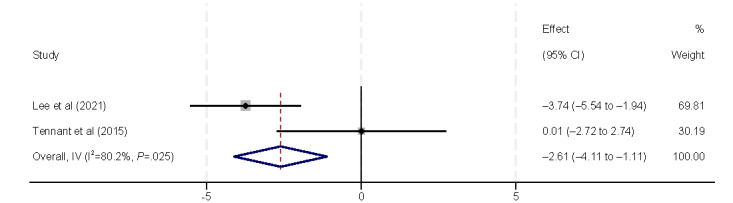
Forest plot of the meta-analytic association between ethnicity and eHealth literacy. IV: inverse variance weighted.

**Figure 8:**
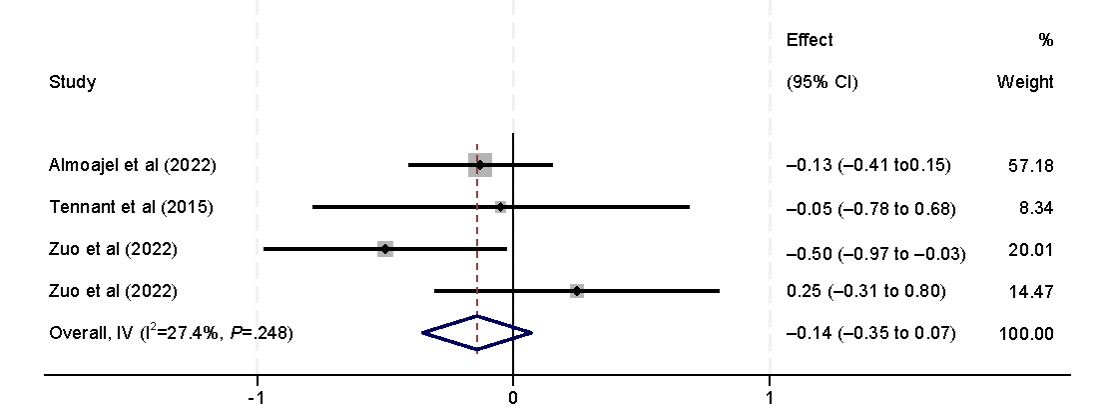
Forest plot of the meta-analytic association between marital status and eHealth literacy. IV: inverse variance weighted.

Ethnicity was also found to affect eHealth literacy (β=–2.61, 95% CI –4.11 to –1.11) [9,41]. Lee et al [41] found that lower eHealth literacy is associated with patients of minority ethnicity (Malaysian Chinese). However, Tennant et al [9] found that ethnicity is not significantly associated with eHealth literacy (Figure 7). Marital status was also included in the analysis (Figure 8). However, no statistically significant association was found, consistent with the results of the 3 (18%) studies [9,32,39].

**Figure 9:**
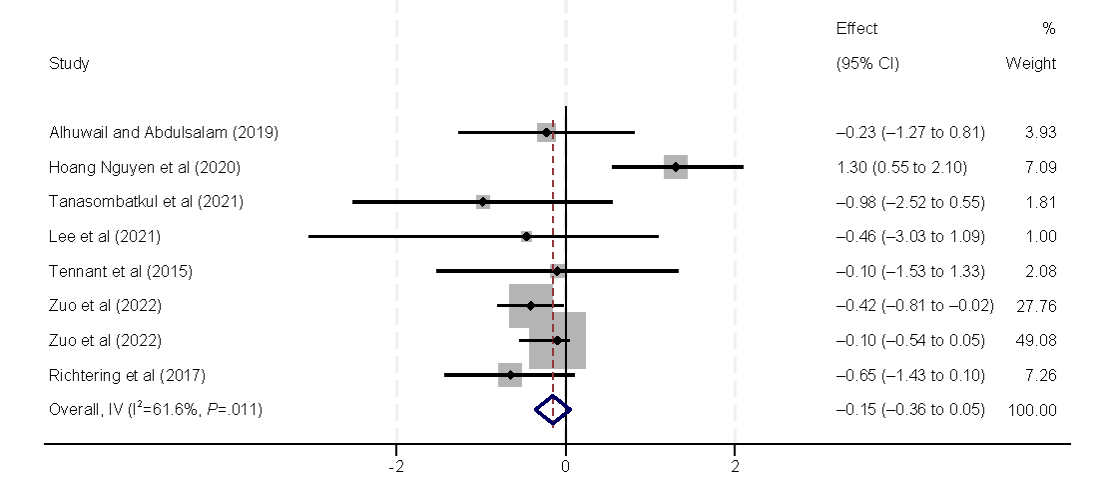
Forest plot of the meta-analytic association between sex and eHealth literacy. IV: inverse variance weighted.

Seven studies [9,29,32,37,38,41,44] assessed the relationship between sex and eHealth literacy using multivariate analysis (Figure 9). Of these, 6 (15%) studies [9,29,32,37,41,44] found that women are at a higher risk of low eHealth literacy than men. However, the final meta-analysis results showed no statistically significant association.

##### Economic and Working Conditions

**Figure 10:**
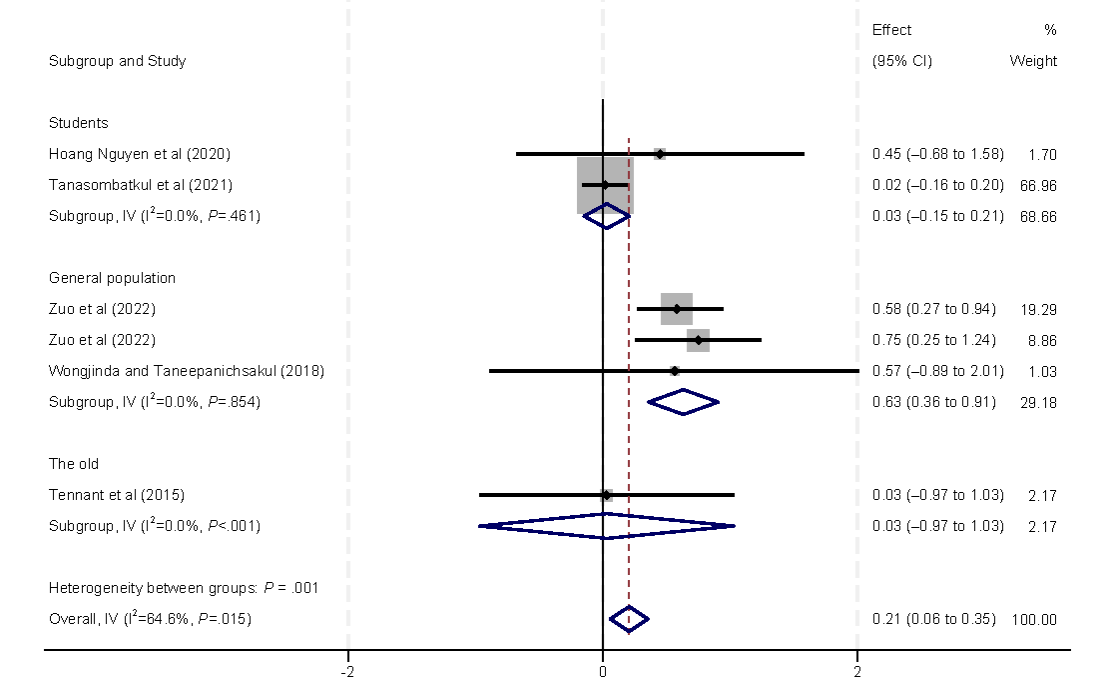
Forest plot of the meta-analytic association between income and eHealth literacy. IV: inverse variance weighted.

**Figure 11:**
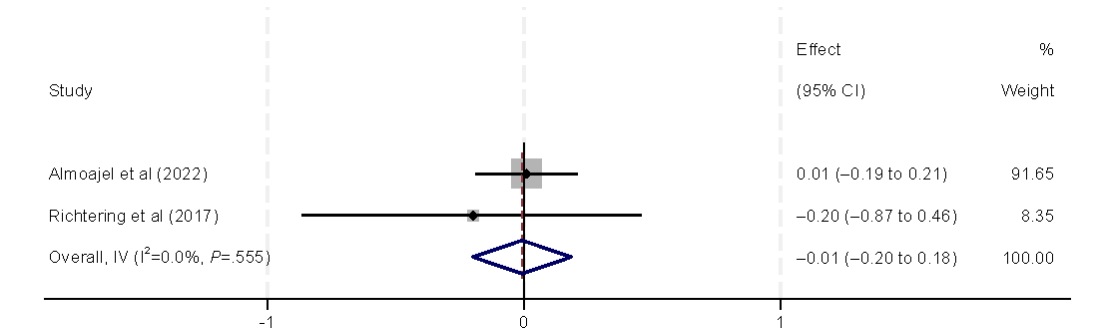
Forest plot of the meta-analytic association between insurance and eHealth literacy. IV: inverse variance weighted.

**Figure 12:**
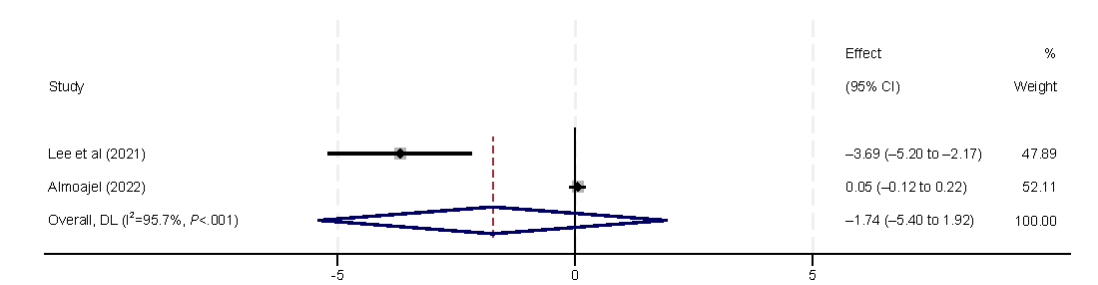
Forest plot of the meta-analytic association between employment status and eHealth literacy. DL: DerSimonian and Laird.

**Figure 13:**
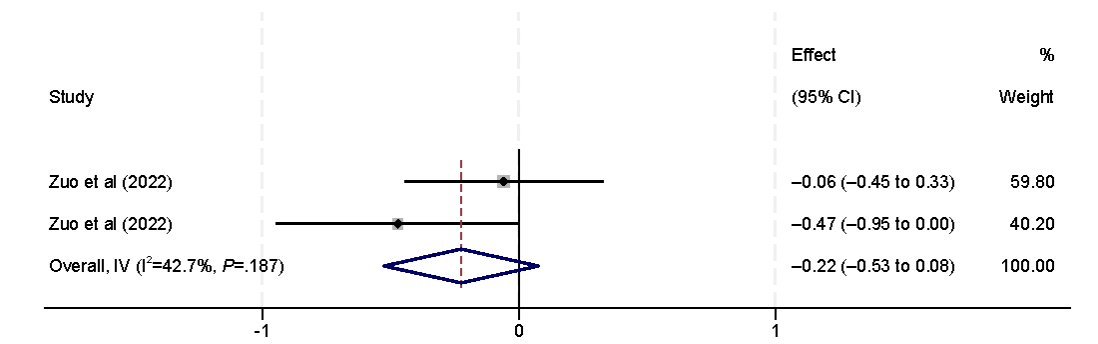
Forest plot of the meta-analytic association between occupation type and eHealth literacy. IV: inverse variance weighted.

Regarding economic and working conditions, we analyzed eHealth literacy in relation to economic aspects (eg, income and insurance coverage), as well as working conditions (eg, employment status and occupation type). All 5 (29%) included studies [9,32,38,40,44] found a positive correlation between income and eHealth literacy (Figure 10), which was also confirmed by the final meta-analysis (β=0.21, 95% CI 0.06-0.35). However, in subgroup analyses, the correlation between income and eHealth literacy was only confirmed in the general population (β=0.63, 95% CI 0.36-0.91). Private insurance is also considered an economic factor. Neither of the 2 (12%) included studies [37,39] found a correlation between the presence or absence of private insurance and the level of eHealth literacy, which aligns with the findings of our meta-analysis (Figure 11). Unemployment status was identified as a risk factor for eHealth literacy levels by Almoajel et al [39] and Lee et al [41]. However, our meta-analysis revealed substantial heterogeneity, which prevented reliable statistical inferences (Figure 12). Zuo et al [32] observed variations in eHealth literacy according to occupation type, but these findings were not supported by the final results of our meta-analysis (Figure 13).

##### Education

**Figure 14:**
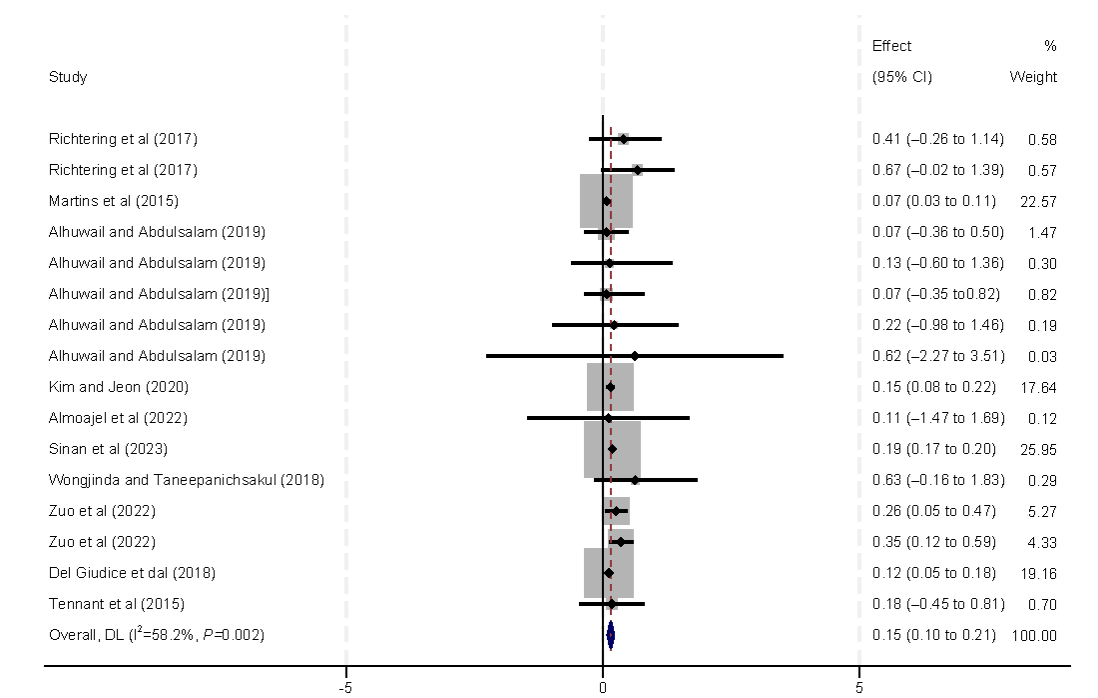
Forest plot of the meta-analytic association between education and eHealth literacy. DL: DerSimonian and Laird.

As shown in Figure 14, 10 (59%) studies [9,29,31-33,37,39,40,42,45] identified lower educational status or a lack of formal education as being associated with lower eHealth literacy (β=0.15, 95% CI 0.10-0.21).

##### Personal Capabilities

**Figure 15:**
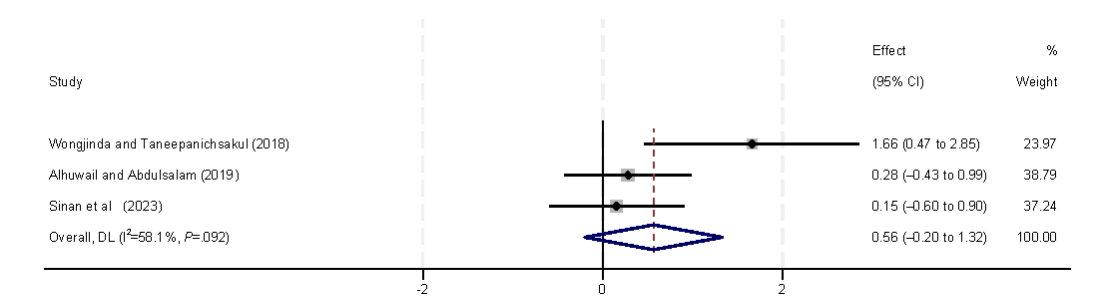
Forest plot of the meta-analytic association between perceived importance and eHealth literacy. DL: DerSimonian and Laird.

**Figure 16:**
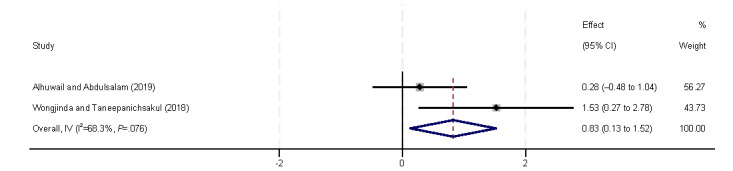
Forest plot of the meta-analytic association between perceived usefulness and eHealth literacy. IV: inverse variance weighted.

**Figure 17:**
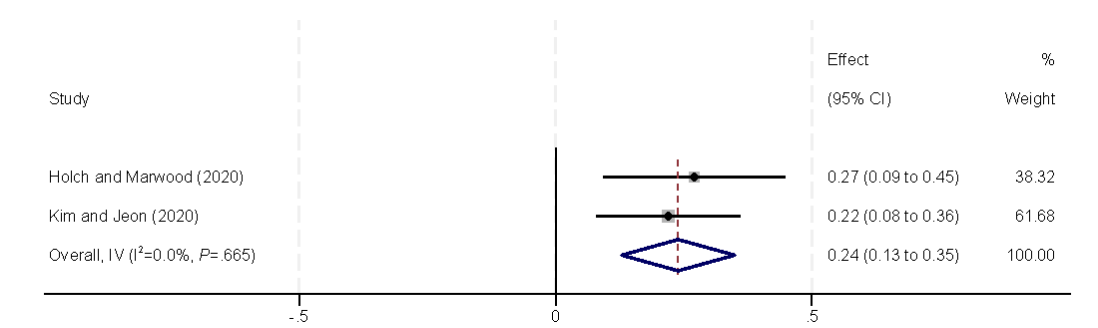
Forest plot of the meta-analytic association between self-efficacy and eHealth literacy. IV: inverse variance weighted.

The personal capabilities of perceived importance, perceived usefulness, and self-efficacy were found to significantly influence eHealth literacy (Figures 15-17). Perceived importance [29,31,40], perceived usefulness (β=0.83, 95% CI 0.13-1.52) [29,40], and self-efficacy (β=0.24, 95% CI 0.13-0.35) [42,43] were positively correlated with eHealth literacy levels in all included studies.

#### Influencing Factors in the Health Status Theme

**Figure 18:**
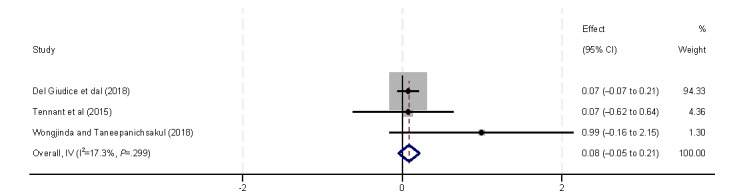
Forest plot of the meta-analytic association between self-rated health and eHealth literacy. IV: inverse variance weighted.

**Figure 19:**
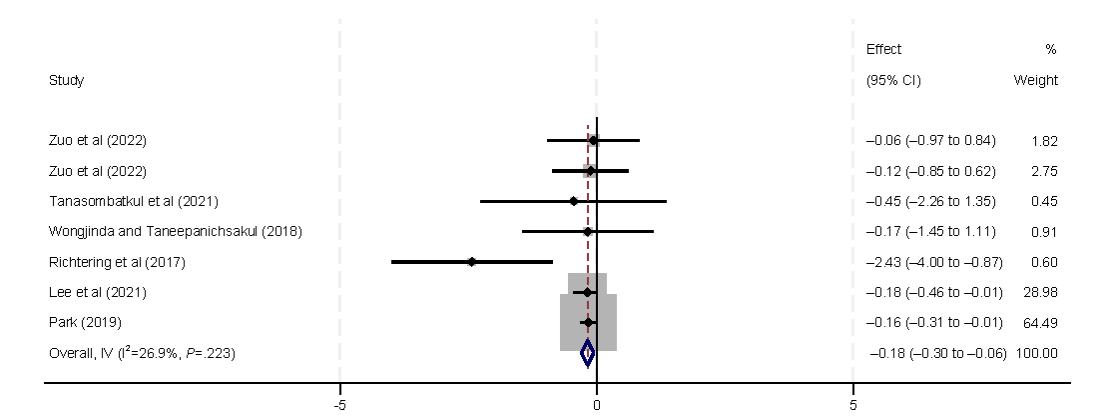
Forest plot of the meta-analytic association between disease and eHealth literacy. IV: inverse variance weighted.

Health status, specifically self-rated health and disease status, was found to significantly influence eHealth literacy (Figures 18 and 19). According to Del Giudice et al [33], Tennant et al [9], and Wongjinda and Taneepanichsakul [40], individuals with better self-rated health exhibit higher levels of eHealth literacy. The 6 (35%) included studies [32,34,37,40,41,44] found that participants with existing disease or at a higher risk for disease have lower levels of eHealth literacy (β=–0.15, 95% CI –0.8 to –0.16).

### Meta-Regression Analyses to Examine Cofounders Affecting eHealth Literacy

In the meta-regression analysis of confounding factors, only age grouping significantly moderated the relationship between income and eHealth literacy (Table 3). For other influencing factors, neither age nor the developmental level of the country where the study was conducted was identified as a significant confounding factor.

**Table 3:** Confounder analysis results.

Confounder and influencing factors			β coefficient (SE)		*t* test (*df*)		*P* value	*I*^2^ (%)		*τ* ^2^
**Age group**										
	Internet usage^a^	0.06 (0.13)		0.49 (7)		.64		67.0	0.072	
	Sex	–0.323 (–0.78)		0.42 (6)		.69		70.2	0.397	
	Income^a^	0.60 (0.17)		3.63 (4)		.04^b^		100.0	0	
	Marital status	0.09 (0.51)		0.19 (2)		.87		50.8	0.0481	
	Education^a^	–0.018 (0.019)		–0.98 (14)		.35		0	0.001	
	Disease^a^	–0.91 (0.37)		–2.48 (5)		.07		0	0	
**Country**										
	Internet usage^a^	0.08 (0.22)		0.37 (7)		.73		67.0	0.072	
	Sex	0.18 (1.53)		0.12 (6)		.91		70.2	0.397	
	Income^a^	–1.20 (–0.59)		2.05 (4)		.13		100.0	0	
	Marital status	0.09 (0.51)		0.19 (2)		.87		50.8	0.04814	
	Education^a^	–0.07 (0.03)		–2.10 (14)		.06		0	0.001	
	Disease^a^	–0.89 (0.37)		–2.35 (5)		.08		0	0	

^a^Significant in meta-analyses.

^b^Significant *P* value.

## Discussion

### Principal Findings

We summarized and analyzed the findings from relevant scientific studies, highlighting significant variability in the reported factors influencing eHealth literacy across different study populations. Our meta-analysis indicated that the majority of influencing factors are demographic variables (eg, age, ethnicity) and social determinants of health (eg, income, education). Additionally, we identified factors amenable to clinical intervention, such as internet usage, self-efficacy, and the perceived usefulness of eHealth. However, most of these factors did not achieve a high-level grade according to the GRADE framework.

#### Influencing Factors of eHealth Literacy

Numerous cross-sectional studies have identified various influencing factors of eHealth literacy. This study categorized the influencing factors into 3 main themes based on the Literacy and Health Conceptual Framework and TAM. In the *actions* theme, this study revealed that internet usage is positively associated with eHealth literacy. A possible explanation is that eHealth literacy encompasses an individual’s comprehensive ability to seek, discover, understand, evaluate, and apply health information obtained through electronic channels to address health-related issues. According to the eHealth literacy Lily model, individuals who use the internet more frequently tend to exhibit higher levels of information literacy, media literacy, and computer literacy [15]. This enables them to find and access health information through electronic channels more easily, thereby enhancing their eHealth literacy. Furthermore, some studies have identified a significant negative correlation between computer use stress and eHealth literacy among users [47]. Additionally, users with higher internet usage frequency tend to exhibit stronger self-efficacy in using eHealth technologies, which may reduce their computer use stress and, consequently, enhance their ability to access and use internet health information and services [48]. Recent studies have also confirmed that eHealth literacy can be improved by internet use interventions [49]. Therefore, it is beneficial for health care professionals to provide health education that enhances internet skills, such as training in searching for and evaluating health information.

In the *determinants* theme, various social determinants of health and other complex demographic factors are included. Factors such as age, ethnicity, economic status, and education are closely associated with eHealth literacy [9,14]. The effect of age varies across different population subgroups. Among student groups, eHealth literacy is positively correlated with age, whereas in the general population and among older adults, the correlation is negative. According to socioemotional selectivity theory [50], individual needs change with age. Young people tend to engage in growth-related activities, such as accumulating knowledge and advancing their careers [51]. Consequently, students are typically more active in digital environments, due to both their educational settings and their personal interests, which increasingly leverage information technology for learning and communication [52]. This constant interaction likely enhances their eHealth literacy as they progress through their academic careers. As people grow older, their values shift toward meaning and social needs [51]. When users are not yet aware of their health-related needs, they are less likely to proactively seek health information, hindering actions that could enhance their eHealth literacy. Additionally, research has shown that with aging, especially in the older adult phase, cognitive and physiological decline makes it more challenging to perceive and manage eHealth-related technologies [53]. This reduced ease of use can result in lower adoption of eHealth technologies, making it challenging to improve eHealth literacy levels. Other social determinants of health, such as ethnicity, economic status, and education, contribute to this issue in complex and often unmodifiable ways, closely intertwined with the digital divide [9]. A survey conducted by Choi and Dinitto [6] revealed that low-income and older populations exhibit significantly lower levels of eHealth literacy and internet usage compared to the general population in the United States. This disparity can be attributed to factors such as limited access to computers and internet technologies, financial constraints that prevent the acquisition of personal devices, and limitations caused by medical conditions and disabilities [6]. Modifiable factors that can be targeted for intervention include self-efficacy [54] and the perceived usefulness of eHealth. Self-efficacy reflects an individual’s confidence in the ability [55], and eHealth literacy is defined as an ability [3]. It is reasonable to hypothesize a positive correlation between self-efficacy and eHealth literacy. Moreover, self-efficacy explains the eHealth-adopting motivation, behaviors, and awareness of appraisal and application skills [56]. Perceived usefulness is significantly correlated with eHealth literacy, even as a supplemental question for eHEALS [29,30,37]. This relationship can be explained by TAM [18]. Perceived usefulness is one of the core beliefs that determines individuals’ intention to use IT, defined as the extent to which a person believes that using IT will enhance their performance [17]. Some researchers have proved that individuals with high perceived usefulness may have a more active attitude toward eHealth use [57,58]. In summary, the association between eHealth literacy and background attributes is difficult to modify. However, meso- and macrolevel approaches can be used. At the macro level, these include the level of government support and policy direction [16], for instance, providing technology subsidies or allowances to low-income individuals. Government agencies and nongovernmental organizations (NGOs) can play a significant role in recycling and refurbishing unused or discarded computers for older adults and low-income individuals. These initiatives can be cost-effective, enabling individuals to live more independently, reducing their reliance on both formal and informal support systems, and enhancing their quality of life [6]. The meso level encompasses various characteristics related to the planning, design, and development of eHealth services [16]. Emphasizing user-friendliness is essential. For example, recommendations including simplifying operational interfaces and steps optimize workflows, create more visually prominent interfaces, provide clearer system prompts, and replace technical terms with plain language and illustrations in designs tailored for older adults [57]. From an individual’s micro perspective, interventions can focus on enhancing self-efficacy and perceived usefulness. Older adults who are hesitant to use the internet due to low self-efficacy in technology can be encouraged through demonstrations and educational interventions. Younger individuals with strong computer and internet skills can assist by volunteering or being employed to teach older adults how to engage with social media, browse the web, and participate in health-related activities. Particularly for low-income, homebound older adults who have limited exposure to computers and internet technology but substantial needs, it is important to emphasize the multiple benefits of internet use and to provide resources, such as equipment and training [6].

In the *health status* theme, disease was identified as a negative influencing factor for eHealth literacy [37,40,41,44]. Previous research indicates that individuals with higher eHealth literacy often achieve better health outcomes [12,59]. However, the relationship between eHealth literacy and health outcomes is complex and not strictly linear. Our study suggests a substantial overlap between populations with low eHealth literacy and those experiencing poor health conditions. Both groups are influenced by social determinants of health and are more likely to be older, less educated, and of a lower socioeconomic status. Additionally, various studies have identified mediating factors in the relationship between health outcomes and eHealth literacy. For example, a cross-sectional study by Li et al [60] involving 802 Chinese residents revealed that social media use mediates the relationship between eHealth literacy and health-promoting behaviors. Similarly, Rabenbauer and Mevenkamp’s [61] study of 224 Australian patients with chronic back pain identified self-efficacy as a mediator in the relationship between eHealth literacy and healthy habits. Therefore, further research is needed to clarify the relationship between eHealth literacy and health outcomes, particularly by exploring this connection after controlling for confounding factors. Future research could also apply health- and literacy-related theories to investigate whether additional factors moderate this relationship.

### Limitations

This study has several limitations that should be acknowledged. First, only studies using the eHEALS measurement tool were included in this review. Insufficient data from other research instruments, such as the eHLQ and the Digital Health Literacy Questionnaire (DHLQ), prevented a comprehensive synthesis of findings across different tools. Second, papers written in languages other than English and Chinese were excluded from this review, potentially introducing language and publication bias. Third, due to the limited number of randomized controlled trials (RCTs) and the inability to combine their effect sizes, RCTs were not included in the meta-analyses. This exclusion is a significant limitation, as RCTs are considered the gold standard for establishing causal relationships. Their exclusion means that the meta-analysis relied more heavily on observational studies, which are more susceptible to confounding factors and bias. This reliance can limit the ability to draw firm conclusions about the causal relationship between eHealth literacy and health outcomes. Fourth, the standardized beta coefficient (β) was used as the effect size measure for data integration in this study, allowing for the inference of correlation relationships but not supporting causal inference. Moreover, the majority of studies included in the meta-analysis focused on student and adolescent populations, which limits the generalizability of the findings to broader populations. Additionally, some of the results reported *I*^2^ exceeding 50%, indicating substantial heterogeneity. This high level of heterogeneity is expected, as eHealth literacy is significantly influenced by social and demographic characteristics, as well as variability in the measurement of influencing factors. This heterogeneity limits the generalizability of the identified factors influencing eHealth literacy. Therefore, the results of the meta-analysis and meta-regression should be interpreted with caution. Finally, all studies included in this research categorized gender using binary terms. This approach overlooks the experiences and outcomes of nonbinary and transgender individuals, potentially leading to a less comprehensive understanding of how eHealth literacy impacts various gender identities. We encourage future research to explore whether differences exist.

### Conclusion

Based on the Literacy and Health Conceptual Framework, we explored the impact of various factors on users’ eHealth literacy across 3 dimensions: actions, determinants, and health status. We found that users’ eHealth literacy is influenced by various factors, including internet usage, age, ethnicity, income, employment status, education, perceived usefulness, self-efficacy, and disease. These findings provide valuable guidance for designing interventions to enhance eHealth literacy.

## Appendix

Multimedia Appendix 1 PRISIMA (Preferred Reporting Items for Systematic Reviews and Meta-Analyses) checklist.

Multimedia Appendix 2 Detailed search strategies for different databases.

Multimedia Appendix 3 Critical appraisal of methodological quality.

Multimedia Appendix 4 Details of studies included for meta-analysis (N=17).

## Abbreviations

DL: DerSimonian and Laird
eHEALS: eHealth Literacy Scale
eHLQ: eHealth Literacy Questionnaire
GRADE: Grading of Recommendations, Assessment, Development, and Evaluation
IV: inverse variance weighted
JBI: Joanna Briggs Institute
PRISMA: Preferred Reporting Items for Systematic Reviews and Meta-Analyses
RCT: randomized controlled trial
TAM: Technology Acceptance Model
