# Supplementary material for: Factors Influencing eHealth Literacy Worldwide: Systematic Review and Meta-Analysis
Source: J Med Internet Res. 2025 Mar 10;27:e50313. doi: 10.2196/50313 (PMC11933766; doi:10.2196/50313)
Supplement: Multimedia Appendix 2 [file jmir_v27i1e50313_app2.docx]

| Pubmed | #1 eHealth Literacy[Title/Abstract] OR Digital Health Literacy[Title/Abstract] OR Electronic health literacy[Title/Abstract] OR e-health literacy[Title/Abstract] OR online health literacy[Title/Abstract] OR telehealth literacy[Title/Abstract] OR Health eLiteracy[Title/Abstract]  #2 influenc*[Title/Abstract]  #3 1 AND 2··········································144 |
| --- | --- |
| Embase | #1 'ehealth literacy'/exp  #2 'ehealth literacy':ab,ti OR 'digital health literacy':ab,ti OR 'electronic health literacy':ab,ti OR 'e-health literacy':ab,ti OR 'online health literacy':ab,ti OR 'telehealth literacy':ab,ti OR 'health eliteracy':ab,ti  #3 1 OR2  #4 influenc*:ab,ti  #5 #3 AND #4···································143 |
| WOS | (TS=(eHealth Literacy OR Digital Health Literacy OR Electronic health literacy OR e-health literacy OR online health literacy OR telehealth literacy OR Health eLiteracy)) AND TS=(influenc*) ················· ·····1054 |
| Cochrane Library | #1 (eHealth Literacy OR Digital Health Literacy OR Electronic health literacy OR e-health literacy OR online health literacy OR telehealth literacy OR Health eLiteracy):ti,ab,kw  #2(influenc*):ti,ab,kw  #3 #1 AND #2·····································67 |
| Medline | #1(eHealth Literacy or Digital Health Literacy or Electronic health literacy or e-health literacy or online health literacy or telehealth literacy or Health eLiteracy).ab,ti.  #2 "influenc*".ab,ti.  #3 #1 AND#2·····································133 |
| CINAHL | #1TI ( eHealth Literacy OR Digital Health Literacy OR Electronic health literacy OR e-health literacy OR online health literacy OR telehealth literacy OR Health eLiteracy ) OR AB ( eHealth Literacy OR Digital Health Literacy OR Electronic health literacy OR e-health literacy OR online health literacy OR telehealth literacy OR Health eLiteracy )  #2 TI influenc* OR AB influenc*  #3 #1 AND #2··································264 |
| CNKI | #1 电子健康素养  #2 影响因素  #3 #1 AND #2··································143 |

eHealth Literacy OR Digital Health Literacy OR Electronic health literacy OR e-health literacy OR online health literacy OR telehealth literacy OR Health eLiteracy
