# Supplementary material for: Factors Influencing eHealth Literacy Worldwide: Systematic Review and Meta-Analysis
Source: J Med Internet Res. 2025 Mar 10;27:e50313. doi: 10.2196/50313 (PMC11933766; doi:10.2196/50313)
Supplement: Multimedia Appendix 3 [file jmir_v27i1e50313_app3.docx]

The quality assessment appraisal was performed by two independent assessors using the standardized Joanna Briggs Institute (JBI) critical appraisal tool prepared for cross-sectional studies and cohort studies. The tools have ‘Yes’, ‘No’, ‘Unclear’ or ‘not applicable’ responses, and scores were given 1 for ‘Yes’, 0 for ‘No’ and ‘Unclear’ responses. Scores for each item were summed up and transformed into percentages. The average score of the two independent assessors were taken. Only studies that scored ≥50% were considered for systematic review and meta-analysis.

1. **JBI Critical Appraisal Checklist for Analytical cross-sectional studies**

1. Were the criteria for inclusion in the sample clearly defined?

2. Were the study subjects and the setting described in detail?

3. Was the exposure measured in a valid and reliable way?

4. Were objective, standard criteria used for measurement of the condition?

5. Were confounding factors identified?

6. Were strategies to deal with confounding factors stated?

7. Were the outcomes measured in a valid and reliable way?

8. Was appropriate statistical analysis used?

Based on the above criteria, the quality score for analytical cross-sectional studies is given below.

| Author | Criteria and corresponding scores | | | | | | | | Total | % |
| --- | --- | --- | --- | --- | --- | --- | --- | --- | --- | --- |
|  | **#1** | **#2** | **#3** | **#4** | **#5** | **#6** | **#7** | **#8** |  |  |
| Alhuwail and Abdulsalam [1] | 1 | 1 | 1 | 1 | 0 | 1 | 1 | 1 | 7 | 87.5 |
| Almoajel et al [2] | 1 | 1 | 1 | 1 | 0 | 1 | 1 | 1 | 7 | 87.5 |
| Gazibara et al [3] | 1 | 1 | 1 | 1 | 0 | 1 | 1 | 1 | 7 | 87.5 |
| Ghazi et al [4] | 1 | 1 | 1 | 1 | 1 | 1 | 1 | 1 | 8 | 100 |
| Del Giudice et al [5] | 1 | 1 | 1 | 1 | 0 | 0 | 1 | 1 | 6 | 75 |
| Holch and Marwood [6] | 1 | 0 | 1 | 1 | 0 | 1 | 1 | 1 | 6 | 75 |
| Kim and Jeon [7] | 1 | 1 | 1 | 1 | 1 | 1 | 1 | 1 | 8 | 100 |
| Hoang Nguyen and Bich Thi Le [8] | 1 | 1 | 1 | 1 | 0 | 1 | 1 | 1 | 7 | 87.5 |
| Lee et al [9] | 1 | 1 | 1 | 1 | 1 | 1 | 1 | 1 | 8 | 100 |
| Martins et al [10] | 0 | 0 | 1 | 1 | 0 | 1 | 1 | 1 | 5 | 62.5 |
| Park et al [11] | 1 | 1 | 1 | 1 | 1 | 1 | 1 | 1 | 8 | 100 |
| Richtering et al [12] | 1 | 1 | 1 | 1 | 1 | 1 | 1 | 1 | 8 | 100 |
| Sinan et al [13] | 1 | 1 | 1 | 1 | 1 | 1 | 1 | 1 | 8 | 100 |
| Tanasombatkul et al [14] | 0 | 1 | 1 | 1 | 1 | 1 | 1 | 1 | 7 | 87.5 |
| Tennant et al [15] | 1 | 1 | 1 | 1 | 0 | 0 | 1 | 1 | 6 | 75 |
| Wongjinda et al [16] | 0 | 1 | 1 | 1 | 1 | 1 | 1 | 1 | 7 | 87.5 |
| Zuo et al [17] | 1 | 0 | 1 | 1 | 1 | 1 | 1 | 1 | 7 | 87.5 |

## References

1. Alhuwail D, Abdulsalam Y. Assessing electronic health literacy in the state of Kuwait: survey of internet users from an Arab state. *J Med Internet Res* 2019 May 24;21(5):e11174.
2. Almoajel A, Alshamrani S, Alyabsi M. The relationship between e-health literacy and breast cancer literacy among Saudi women. *Front Public Health* 2022;10:841102.
3. Gazibara T, Cakic M, Cakic J, Grgurevic A, Pekmezovic T. Familiarity with the internet and health apps, and specific topic needs are amongst the factors that influence how online health information is used for health decisions amongst adolescents. *Health Info Libr J* 2022 Jun 2.
4. Ghazi SN, Berner J, Anderberg P, Sanmartin Berglund J. The prevalence of eHealth literacy and its relationship with perceived health status and psychological distress during Covid-19: a cross-sectional study of older adults in Blekinge, Sweden. *BMC Geriatr* 2023 Jan 4;23(1):5.
5. Del Giudice P, Bravo G, Poletto M, De Odorico A, Conte A, Brunelli L, Arnoldo L, Brusaferro S. Correlation between eHealth literacy and health literacy using the eHealth Literacy Scale and real-life experiences in the health sector as a proxy measure of functional health literacy: cross-sectional web-based survey. *J Med Internet Res* 2018 Oct 31;20(10):e281.
6. Holch P, Marwood JR. EHealth literacy in UK teenagers and young adults: exploration of predictors and factor structure of the eHealth Literacy Scale (eHEALS). *JMIR Form Res* 2020 Sep 8;4(9):e14450.
7. Kim S, Jeon J. Factors influencing eHealth literacy among Korean nursing students: a cross-sectional study. *Nurs Health Sci* 2020 Sep;22(3):667–674.
8. Hoang Nguyen L, Bich Thi Le T. E-health literacy of medical students at a university in Central Vietnam. *Indian J Public Health Res Dev* 2020 Feb 1;11(2):1299. doi: 10.37506/v11/i2/2020/ijphrd/195001
9. Lee WL, Lim ZJ, Tang LY, Yahya NA, Varathan KD, Ludin SM. Patients’ technology readiness and eHealth literacy: implications for adoption and deployment of eHealth in the COVID-19 era and beyond. *Comput Inform Nurs* 2021 Nov 2;40(4):244–250.
10. Martins A, Andrade I, Pocinho R, Belo P. e-Health literacy in ageing. Proceedings of the 3rd International Conference on Technological Ecosystems for Enhancing Multiculturality Porto Portugal; 2015. pp. 53–57. doi: 10.1145/2808580.2808589
11. Park BK. Factors Influencing eHealth literacy of middle school students in Korea: a descriptive cross-sectional study. *Healthc Inform Res* 2019 Jul;25(3):221–229.
12. Richtering SS, Hyun K, Neubeck L, Coorey G, Chalmers J, Usherwood T, Peiris D, Chow CK, Redfern J. eHealth literacy: predictors in a population with moderate-to-high cardiovascular risk. *JMIR Hum Factors* 2017 Jan 27;4(1):e4.
13. Sinan O, Ayaz-Alkaya S, Akca A. Predictors of eHealth literacy levels among nursing students: a descriptive and correlational study. *Nurse Educ Pract* 2023 Mar;68:103592.
14. Tanasombatkul K, Pinyopornpanish K, Angkurawaranon C, Buawangpong N, Rojanasumapong A, Jiraporncharoen W. Is Electronic health literacy associated with learning outcomes among medical students in the first clinical year?: a cross-sectional study. *Eur J Investig Health Psychol Educ* 2021 Aug 19;11(3):923–932.
15. Tennant B, Stellefson M, Dodd V, Chaney B, Chaney D, Paige S, Alber J. eHealth literacy and Web 2.0 health information seeking behaviors among baby boomers and older adults. *J Med Internet Res* 2015 Mar 17;17(3):e70.
16. Wongjinda LT, Taneepanichsakul S. Determinants of eHealth literacy level among Royal Thai Army personnel: a case. *Southeast Asian J Trop Med Public Health* 2018;49(1).
17. Zuo Q, Cheng J, Peng W, Zhu L, Jiang X. Differences analysis of community residents' e-Health literacy level and influencing factors between urban and rural. *Chin Nurs Res* 2022;36(4):587–593.
