# Supplementary material for: Factors Influencing eHealth Literacy Worldwide: Systematic Review and Meta-Analysis
Source: J Med Internet Res. 2025 Mar 10;27:e50313. doi: 10.2196/50313 (PMC11933766; doi:10.2196/50313)
Supplement: Multimedia Appendix 4 [file jmir_v27i1e50313_app4.docx]

**Table S1.** Details of studies included for meta-analysis (N=17).

| Study | Country | Sample size, N | Study population | Age (years), mean (SD) | Funding | eHealth literacy status^a^, mean (SD) | Extracted determinants (from multivariable model) | Summary |
| --- | --- | --- | --- | --- | --- | --- | --- | --- |
| Sinan et al [30] | Turkey | 1059 | Nursing students at nursing departments of 2 state universities in Ankara, Turkey | 21.14 (1.62) | NR^b^ | 29.28 (4.73) | Academic level, gender, income, place of residence, perception of health, chronic disease, daily internet use duration, search health-related information on the internet, frequency of internet use, find accessing health resources on the internet important, find the internet useful when making health decisions, watch video/film on the internet, digital game playing on the internet, social media use | Fourth-year students (β=.186) had higher eHealth literacy scores than those in the first, second, and third years. Students who used the internet frequently and always (β=.116), those who searched for health-related information on the internet (β=.093), those who found accessing health information on the internet important (β=.153), those who found the internet useful when making health decisions (β=.153), and those who played digital games on the internet (β=.062) had significantly higher eHealth literacy scores (*P*<.05). |
| Zuo et al [32] | China | 961 | Adult residents of urban and rural communities | NR | NR | 32(24,40)^c^, 28 (21.34)^d^ | Gender, age, education level, marital status, whether suffering from chronic diseases, family economic status, occupation | Gender (OR^e^ 0.66, 95% CI 0.446-0.976), literacy (OR 1.298, 95% CI 1.053-1.601), marital status (OR 0.607, 95% CI 0.378-0.974), and household economic status (OR 0.577, 95% CI 0.395-0.844) had a statistically significant effect on eHEALS scores of residents of rural communities. The effects of literacy (OR 1.426, 95% CI 1.126-1.806) and household economic status (OR 0.472, 95% CI 0.288-0.775) on eHEALS scores of residents in urban communities were statistically significant. |
| Del Giudice et al [33] | Italy | 868 | Undergraduate and postgraduate students | NR | NR | 28.2 (6.2) | Age, educational attainment, self-rated health, frequency of internet use for health | Our study findings show that the IT-eHEALS scores have a weak, positive correlation with age (*P*=.002), educational attainment (*P*=.001), and self-rated health (*P*=.038). |
| Park [34] | South Korea | 784 | Middle school students | NR | Kyungpook National University Research Fund in 2017 | 28.72 (5.68) | Grade, number of hospital admissions, number of diseases diagnosed, asthma, herniated disc, atopic dermatitis | Third-year middle school grade (β=.137, *P*=.001), second-year middle school grade (β=.091, *P*=.022), and number of diseases diagnosed (β=−.159, *P*=.001) were significant predictors of eHealth literacy among middle school students. |
| Gazibara et al [35] | Serbia | 702 | Adolescents aged 14-19 years | NR | Ministry of Science and Technological Development of the Republic of Serbia (grant/award 175087) | 26.0 (10.0) | Gender, age, school program, grade point average, monthly income, parental education, age at first internet use | Parental education (β=.27, *P*=.001) was a significant predictor of eHealth literacy. |
| Ghazi et al [36] | Sweden | 490 | Older adults as participants of the Swedish National Study on Aging and Care, Blekinge (SNAC-B) | 78 (2.4) | Open access funding provided by the Blekinge Institute of Technology | 27.5 (10.8) | Gender, age, psychological distress | Being female (β=.01, *P*=.075) and age (β=–.04, *P*<.001) were associated with high eHealth literacy (*P*<.05). |
| Richtering et al [37] | Australia | 453 | Adults with moderate-to-high risk for a CVD^f^ event | 67 (8.0) | National Health and Medical Research Council (NHMRC) Project (grant APP1047508) | 27.2 (6.59) | Age, education level, income, private insurance, polypharmacy, history of coronary disease, time spent on the internet, main device used to access the internet | After adjustment for demographic, socioeconomic, and technology use, only the time spent on the internet (*P*=.01) was associated with the level of eHealth literacy. Participants who spent less than or equal to 1 hour on the internet per day were 2.45 times more likely to have low eHealth literacy compared to those who spent more than 1 hour per day. Conversely, age (*P*=.26), gender (*P*=.18), education (*P*=.19), income (*P*=.15), CVD-related polypharmacy (*P*=.22), private insurance (*P*=.47), and the main device used to access the internet (*P*=.30) did not achieve statistical significance. |
| Hoang Nguyen et al [38] | Vietnam | 410 | Medical students | NR | NR | 27.03 (3.54) | Age, sex, economic condition, training program, learning classification, number of foreign languages studied, English level, computer skills, own a facility to access the internet, purpose of seeking electronic health information (to look for a solution to health problem, to respond to learning requests, to improve knowledge about health and professionals) | Factors influencing eHealth literacy were gender (*P*=.001), training program (*P*=.013), computer skills (*P*=.031), and purpose of seeking and using medical information (*P*<.001). |
| Alhuwail and Abdulsalam [28] | Kuwait | 386 | General population | NR | NR | 28.63 (5.6) | Intercept, usefulness, importance, gender, age, education, internet, usage | Males had a lower eHEALS score than females (β=−.23, *P*<.05); individuals with a doctorate degree were expected to have a higher eHEALS score compared to bachelor’s degree holders by an average of 3.5 points; individuals who used the internet more than 5 hours a day on average were expected to have a higher eHEALS score compared to individuals who used the internet an average of 1-3 hours daily. |
| Almoajel et al [39] | Saudi Arabia | 336 | General population | 24 (1.48) | NR | 28.79 (6.75) | Age, education level, marital status, number of children, employment, income, health insurance | Statistically significant predictors of eHealth literacy included age and education. The total eHEALS score decreased by 0.26 points for each year of age increased (β=−.26, *P*=.02). This indicated that on average, the youngest participants (18 years old) were likely to score higher on eHEALS than older adults. In addition, as the education level (β=.11, *P*= 0.04) increased, the total eHEALS scores also increased. Marital status, the number of children, employment status, income, and health insurance status were not significantly associated with eHealth literacy. |
| Wongjinda and Taneepanichsakul [40] | Thailand | 300 | Royal Thai Army (RTA) personnel | NR | College of Public Health Sciences, Chulalongkorn University, Armed Forces Institute of Medical Sciences, Royal Thai Army Medical Department | 31.6 (NR) | Age, occupation, education, income, history of underlying disease, perceived health status, internet use, previously used eHealth, eHealth perceived to be useful or very useful, eHealth perceived to be important or very important | Factors significantly (*P*<.05) associated with eHealth literacy levels were perceived usefulness of the internet to make decisions about health and perceived importance of being able to access health resources on the internet. |
| Tennant et al [9] | United States | 283 | Older adults | 67.46 (9.98) | National Institutes of Health (NIH), National Center for Advancing Translational Sciences (NCATS), Clinical and Translational Science Awards (CTSA) awards to the University of Florida (UL1TR000064 and KL2TR000065) | 29.05 (5.75) | Sex, age, marital status, ethnicity, race, education level, income, health status, total number of electronic devices used to seek health information | As age (β=−.10) increased by 1 year, the total eHEALS score decreased by 0.10 points. This indicated that on average, the youngest baby boomers of age 50 years were likely to score approximately 1.56 points higher on eHEALS than older adults who were 65 years of age. In addition, as the education level (β=.48) increased, the total eHEALS score increased by 0.48 points. Finally, holding all other factors in the regression model constant, the use of more electronic devices to access web-based health information (β=1.26) was significantly associated with greater eHealth literacy. Sex, marital status, race, ethnicity, income, and health status were not significantly associated with eHealth literacy. |
| Lee et al [41] | Malaysia | 276 | General population | 47 (NR) | NR | 27.38 (6.59) | Sex, ethnicity, education, income, work status, cardiovascular risk, using the internet for health-related purposes, age, technology readiness scales, innovativeness, insecurity | Patients with Chinese ethnicity (β=−.289, *P*<.001), those educated up to the secondary level (β=−.141, *P*=.019), and those who had more than 1 cardiovascular risk factor (β=−.178, *P*=.004) were associated with lower eHealth literacy. Patients’ previous use of the internet for health-related purposes (β=.217, *P*=.001) was associated with higher eHealth literacy. The technology readiness, tested in model 2, explained additional 29.5% of the variance in eHealth literacy beyond the effects of sociomedicodemographic variables. All subdomains of technology readiness were significant predictors of eHealth literacy (*P*<.001) except for the “insecurity” subdomain. |
| Kim and Jeon [42] | South Korea | 205 | Nursing students | 21.69 (2.77) | National Research Foundation of Korea (NRF) grant funded by the Korea government (MSIT; grant 2019R1G1A1100177) | 29.68 (NR) | Age, sex, academic level, residential area, self-recognized socioeconomic status, total internet usage period (years), computer-related certifications, ease of internet access, e-learning experience | Participants with high eHealth literacy were those over 22 years of age (t=−2.93, *P*=.004), those with an academic level of nursing senior (*F*=7.21, *P*<.001), those with an internet usage period of over 15 years (*F*=5.79, *P*=.004), and those with easy accessibility (*F*=4.74, *P*=.010). |
| Holch and Marwood [43] | United Kingdom | 188 | Undergraduate students | 20.13 (2.16) | NR | 29.46 (4.91) | Newest Vital Sign (NVS), Need for Cognition Scale, Irrational Health Belief Scale | eHEALS and the General Self-Efficacy Scale (GSE) were significantly positively correlated (r=0.28, *P*<.001), and hierarchical linear modeling revealed the GSE as a significant predictor of scores on eHEALS (*F*_1,186_=16.16, *P*<.001, R^2^=0.08), accounting for 8.0% of the variance. |
| Tanasombatkul et al [44] | Thailand | 88 | Medical students | 22 (NR) | NR | 33.45 (3.28) | Female gender, age, hometown, income, scholarship, highest parent education, parent job as a health care provider, underlying disease, hours of internet use/day, number of devices | There was no significant association between variables and eHEALS scores, except for long-time internet use (coefficient=0.23, 95% CI 0.04-0.42, *P*=.014). |
| Martins et al [45] | Portugal | 86 | Older adults | 67.36 (7.23) | NR | NR | Age, education level, experience in using the internet | Age was a better predictor of eHealth literacy (β=–.609, *P*<.01). |

^a^Measured with the eHealth Literacy Scale (eHEALS).

^b^NR: not reported.

^c^Median (IQR) of eHEALS for rural community residents.

^e^OR: odds ratio.

^d^Median (IQR) of eHEALS for urban community residents.

^f^CVD: cardiovascular disease.

## References

1. Sinan O, Ayaz-Alkaya S, Akca A. Predictors of eHealth literacy levels among nursing students: a descriptive and correlational study. *Nurse Educ Pract* 2023 Mar;68:103592.
2. Zuo Q, Cheng J, Peng W, Zhu L, Jiang X. Differences analysis of community residents' e-Health literacy level and influencing factors between urban and rural. *Chin Nurs Res* 2022;36(4):587–593.
3. Del Giudice P, Bravo G, Poletto M, De Odorico A, Conte A, Brunelli L, Arnoldo L, Brusaferro S. Correlation between eHealth literacy and health literacy using the eHealth Literacy Scale and real-life experiences in the health sector as a proxy measure of functional health literacy: cross-sectional web-based survey. *J Med Internet Res* 2018 Oct 31;20(10):e281.
4. Park BK. Factors Influencing eHealth literacy of middle school students in Korea: a descriptive cross-sectional study. *Healthc Inform Res* 2019 Jul;25(3):221–229.
5. Gazibara T, Cakic M, Cakic J, Grgurevic A, Pekmezovic T. Familiarity with the internet and health apps, and specific topic needs are amongst the factors that influence how online health information is used for health decisions amongst adolescents. *Health Info Libr J* 2022 Jun 2.
6. Ghazi SN, Berner J, Anderberg P, Sanmartin Berglund J. The prevalence of eHealth literacy and its relationship with perceived health status and psychological distress during Covid-19: a cross-sectional study of older adults in Blekinge, Sweden. *BMC Geriatr* 2023 Jan 4;23(1):5.
7. Richtering SS, Hyun K, Neubeck L, Coorey G, Chalmers J, Usherwood T, Peiris D, Chow CK, Redfern J. eHealth literacy: predictors in a population with moderate-to-high cardiovascular risk. *JMIR Hum Factors* 2017 Jan 27;4(1):e4.
8. Hoang Nguyen L, Bich Thi Le T. E-health literacy of medical students at a university in Central Vietnam. *Indian J Public Health Res Dev* 2020 Feb 1;11(2):1299. doi: 10.37506/v11/i2/2020/ijphrd/195001
9. Alhuwail D, Abdulsalam Y. Assessing electronic health literacy in the state of Kuwait: survey of internet users from an Arab state. *J Med Internet Res* 2019 May 24;21(5):e11174.
10. Almoajel A, Alshamrani S, Alyabsi M. The relationship between e-health literacy and breast cancer literacy among Saudi women. *Front Public Health* 2022;10:841102.
11. Wongjinda LT, Taneepanichsakul S. Determinants of eHealth literacy level among Royal Thai Army personnel: a case. *Southeast Asian J Trop Med Public Health* 2018;49(1).
12. Tennant B, Stellefson M, Dodd V, Chaney B, Chaney D, Paige S, Alber J. eHealth literacy and Web 2.0 health information seeking behaviors among baby boomers and older adults. *J Med Internet Res* 2015 Mar 17;17(3):e70.
13. Lee WL, Lim ZJ, Tang LY, Yahya NA, Varathan KD, Ludin SM. Patients’ technology readiness and eHealth literacy: implications for adoption and deployment of eHealth in the COVID-19 era and beyond. *Comput Inform Nurs* 2021 Nov 2;40(4):244–250.
14. Kim S, Jeon J. Factors influencing eHealth literacy among Korean nursing students: a cross-sectional study. *Nurs Health Sci* 2020 Sep;22(3):667–674.
15. Holch P, Marwood JR. EHealth literacy in UK teenagers and young adults: exploration of predictors and factor structure of the eHealth Literacy Scale (eHEALS). *JMIR Form Res* 2020 Sep 8;4(9):e14450.
16. Tanasombatkul K, Pinyopornpanish K, Angkurawaranon C, Buawangpong N, Rojanasumapong A, Jiraporncharoen W. Is Electronic health literacy associated with learning outcomes among medical students in the first clinical year?: a cross-sectional study. *Eur J Investig Health Psychol Educ* 2021 Aug 19;11(3):923–932.
17. Martins A, Andrade I, Pocinho R, Belo P. e-Health literacy in ageing. Proceedings of the 3rd International Conference on Technological Ecosystems for Enhancing Multiculturality Porto Portugal; 2015. pp. 53–57. doi: 10.1145/2808580.2808589
